# Nutritional Assessment of Childhood Cancer Survivors (the Swiss Childhood Cancer Survivor Study-Nutrition): Protocol for a Multicenter Observational Study

**DOI:** 10.2196/14427

**Published:** 2019-11-18

**Authors:** Fabiën Naomi Belle, Maja Beck Popovic, Marc Ansari, Maria Otth, Claudia Elisabeth Kuehni, Murielle Bochud

**Affiliations:** 1 Institute of Social and Preventive Medicine University of Bern Bern Switzerland; 2 Center for Primary Care and Public Health (Unisanté) University of Lausanne Lausanne Switzerland; 3 Pediatric Hematology-Oncology Unit Lausanne University Hospital Centre Hospitalier Universitaire Vaudois Lausanne Switzerland; 4 Pediatric Onco-Hematology Unit Geneva University Hospital Geneva Switzerland; 5 CANSEARCH Research Laboratory Geneva Medical School Geneva Switzerland; 6 Children’s University Hospital of Bern University of Bern Bern Switzerland

**Keywords:** child, cancer survivors, urine specimen collection, diet surveys, food frequency questionnaire, Swiss Childhood Cancer Registry, Switzerland

## Abstract

**Background:**

Childhood cancer survivors are at high risk of developing adverse late health effects. Poor nutritional intake may contribute to this risk, but information about dietary intake is limited.

**Objective:**

This study will assess childhood cancer survivors’ dietary intake and compare two dietary assessment tools: a self-reported food frequency questionnaire, and dietary measurements from urine spot samples.

**Methods:**

In a substudy of the Swiss Childhood Cancer Survivor Study (SCCSS), SCCSS-Nutrition, we assessed childhood cancer survivors’ dietary intake via a validated food frequency questionnaire. We sent a urine spot collection kit to a subset of 212 childhood cancer survivors from the French-speaking region of Switzerland to analyze urinary sodium, potassium, urea, urate, creatinine, and phosphate content. We will compare the food frequency questionnaire results with the urine spot analyses to quantify childhood cancer survivors’ intake of various nutrients. We collected data between March 2016 and March 2018.

**Results:**

We contacted 1599 childhood cancer survivors, of whom 919 (57.47%) returned a food frequency questionnaire. We excluded 11 childhood cancer survivors who were pregnant or were breastfeeding, 35 with missing dietary data, and 71 who had unreliable food frequency questionnaire data, resulting in 802 childhood cancer survivors available for food frequency questionnaire analyses. To a subset of 212 childhood cancer survivors in French-speaking Switzerland we sent a urine spot collection kit, and 111 (52.4%) returned a urine sample. We expect to have the results from analyses of these samples in mid-2019.

**Conclusions:**

The SCCSS-Nutrition study has collected in-depth dietary data that will allow us to assess dietary intake and quality and compare two dietary assessment tools. This study will contribute to the knowledge of nutrition among childhood cancer survivors and is a step toward surveillance guidelines and targeted nutritional recommendations for childhood cancer survivors in Switzerland.

**Trial Registration:**

ClinicalTrials.gov NCT03297034; https://clinicaltrials.gov/ct2/show/NCT03297034

**International Registered Report Identifier (IRRID):**

DERR1-10.2196/14427

## Introduction

### Background

Survival rates among childhood cancer patients have increased markedly and, due to new and improved treatments, now exceed 80% [1]. As patients live longer, strategies to promote long-term overall health of childhood cancer survivors (CCSs) become increasingly important. Complications and disabilities from treatment, such as chemotherapy and radiotherapy, cancer recurrence, or both, can affect morbidity and mortality many years after a cancer diagnosis [1,2]. The St. Jude Lifetime Cohort Study showed that a large proportion of CCSs experience late effects 25 years after diagnosis; 95% have had at least one chronic health condition and 80% have had a severe, life-threatening, or disabling condition [3]. Frequently reported late effects include cardiovascular diseases (CVDs), endocrine disorders, musculoskeletal problems, and secondary malignancies [2]. Such late effects may be increased by lifestyle habits and choices. Accumulating research in CCSs shows that late effects such as type 2 diabetes, metabolic syndrome, and CVD can be reduced through diet adaptations, weight management, and physical activity [4-7]. Nutrition is an important determinant of the health of CCSs.

However, little is known about the dietary habits of CCSs [8,9], and studies have shown that CCSs adhere poorly to dietary recommendations [10-13]. No evidence-based nutritional guidelines exist specifically for CCSs. Nutritional information can be obtained from, for example, self-reported food frequency questionnaires (FFQs) or 24-hour dietary recalls, whereas assays of biochemical indicators—nutrients or their metabolic products—in tissues or fluids, such as nails, feces, blood, and urine, can more directly quantify intake of nutrients [14]. Since self-reported dietary assessment tools are limited by misreporting and recall bias, which can lead to over- or underreporting, results need to be handled with caution [14]. This holds especially true for dietary assessment using FFQs; underestimation of dietary intake in 16 CCSs was greater when measured by the Block FFQ than by repeated 24-hour dietary recalls, validated by the doubly labelled water method [15]. A Canadian study among 80 CCSs showed that an FFQ could correctly rank CCSs according to their dietary intake when comparing it with 3-day food records [16].

The use of 24-hour urine samples to assess alkaline minerals, halide ions, and protein intake can complement self-reported dietary questionnaires, as well as producing nutritional indicators that potentially are more valid than data from questionnaires [14]. But collection of 24-hour urine samples can be a considerable burden for survivors, and it risks bias due to undetected incomplete sample collection and low response rates. Recent research has focused on the utility of estimating 24-hour urinary output from single spot urine samples [17]. These samples are less burdensome for participants and are more easily obtained by researchers, and potential under- or overcollection is irrelevant [14,17]. By adjusting for parameters such as age, sex, height, and weight, and by taking urinary creatinine into account, samples can yield interpretable results [18]. This makes spot urine samples a practical and cost-saving alternative to collection of 24-hour urine samples. To the best of our knowledge, neither spot urine nor 24-hour urine samples have been studied in CCSs to assess dietary intake.

This study will, to our knowledge, for the first time obtain insight into the dietary intake of CCSs from self-reported FFQs and urinary measurements. It will compare the 2 dietary assessment tools and determine whether spot urine collection from CCSs is feasible.

### Objectives

This study will generate detailed data on the diets of Swiss long-term CCSs. The study’s main objective is to compare the self-reported FFQ dietary assessment tool with assays of urine spot samples. This will give us more information about the reliability of the FFQ, the actual dietary intake of CCSs, and potential associations between dietary intake and the occurrence of somatic late effects. A secondary objective is to evaluate this study itself—that is, to determine the response rate, cost, and CCS reactions of the self-reported FFQ and the dietary markers in spot urine of CCSs.

## Methods

### Study Design

This is a multicenter, observational study incorporated into the Swiss Childhood Cancer Survivor Study (SCCSS). The SCCSS is a population-based, long-term follow-up study of all childhood cancer patients registered in the Swiss Childhood Cancer Registry (SCCR [19]) with leukemia, lymphoma, central nervous system tumors, malignant solid tumors, or Langerhans cell histiocytosis diagnosed in Switzerland; who were under the age of 21 years at the time of diagnosis; who survived 5 years or more after the initial diagnosis of cancer; and who were alive at the time of the study [20-22]. This study is registered at clinicaltrials.gov (NCT03297034).

### Eligibility

CCSs were eligible to participate in the SCCSS-Nutrition study if they had childhood cancer diagnosed between 1976 and 2005, completed a baseline SCCSS questionnaire between 2007 and 2013 [20], and were 18 years of age or older at the time of the follow-up survey in 2017. All CCSs who were enrolled in SCCSS-Nutrition received a follow-up questionnaire including an FFQ. CCSs living in the French-speaking part of Switzerland who returned the questionnaire were invited to provide a urine spot sample. Exclusion criteria were being pregnant or lactating at the time of the study, or having missing or implausible dietary intake information reported in the FFQ [23].

### Recruitment

**Figure 1:**
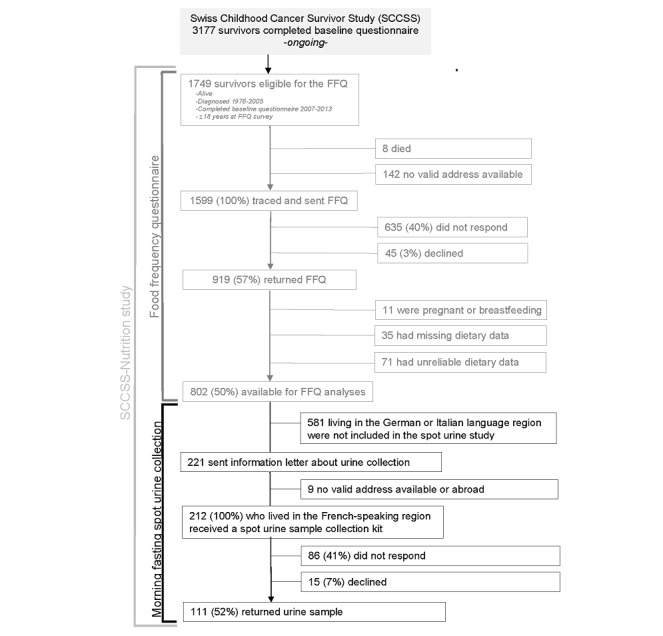
Response rates in the Swiss Childhood Cancer Survivor Study (SCCSS)-Nutrition study. The SCCSS-Nutrition study is subdivided into a food frequency questionnaire assessment (FFQ; gray) and a urine spot collection (black).

**Figure 2:**
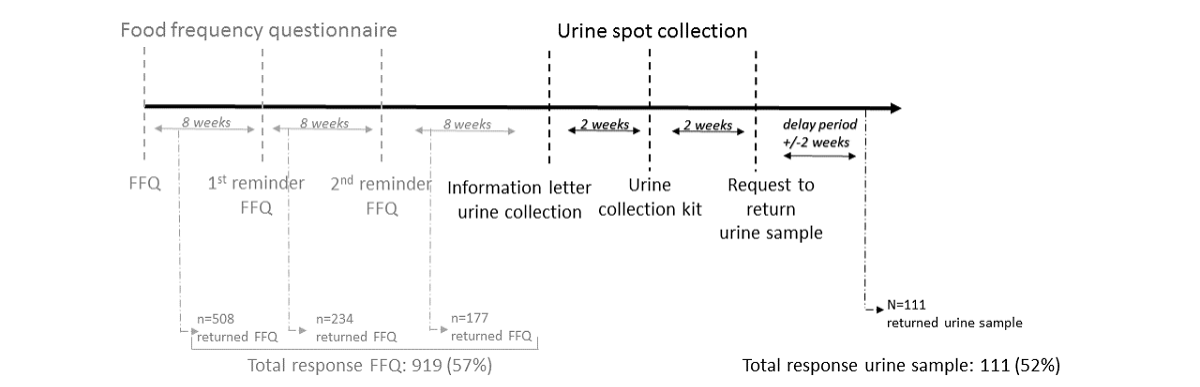
Timelines and response rates of food frequency questionnaire (FFQ) assessment and urine spot collection.

We traced the addresses of all adult CCSs who had completed the baseline questionnaire (n=2527 CCSs) between 2007 and 2013 [20]. Among these, 1749 were 18 years old or older at the time of the survey and thus were eligible for the follow-up questionnaire. In February 2017, we traced 1599 CCSs and sent them a follow-up questionnaire (Figure 1). Nonresponders received a reminder after 8 weeks (Figure 2). If they again did not respond, we sent a second reminder. Finally, 919 (57.47%) CCSs completed the FFQ. We excluded 11 survivors who were pregnant or lactating, 35 who did not report their dietary intake, 71 who had implausible dietary intake data (<850 kcal or >4500 kcal per day) [24], and 581 who lived outside the French-speaking region in Switzerland. We thus sent an information letter signed by the project leader to 221 CCSs who lived in the French-speaking part of Switzerland and asked them for informed consent to provide a urine spot sample. Among these CCSs, 8 were no longer traceable, 1 was abroad, and 15 declined to participate. We sent urine collection kits to the CCSs who agreed to participate and asked them to collect a first morning sample within 2 weeks and post the sample by mail within 24 hours to the pediatric hematology-oncology unit of the University Hospital of Canton Vaud (Centre Hospitalier Universitaire Vaudois [CHUV]; Lausanne, Switzerland). Among these 212 CCS participants, 111 (52.4%) returned a sample. All 111 urine samples met the study protocol and will be available for dietary intake assessment comparison. Those enrolled received no compensation.

### Data Collection

#### Baseline and Follow-Up Questionnaire

From baseline or follow-up questionnaires, we collected CCSs’ data on sex, age at survey, language region in Switzerland in which they lived, country of birth, educational level, living situation, physical activity, smoking status, and height and weight to calculate body mass index. The baseline questionnaire included core questions from the US and UK CCS studies [25,26], with further questions from the Swiss Health Survey and the Swiss census of health-related behaviors and sociodemographic measures [27,28]. The main domains covered by the questionnaire were quality of life, somatic health, fertility, current medication and health services use, psychological distress, health behaviors, and socioeconomic status. The follow-up questionnaire repeated baseline questions on quality of life, somatic health, health behaviors, and socioeconomic status, with the addition of an FFQ to assess dietary intake in detail [29,30].

#### Food Frequency Questionnaire

We assessed CCSs’ dietary intake, including information on portion sizes, with a self-administered, semiquantitative FFQ [31,32] (Multimedia Appendix 1). The FFQ was originally developed and validated against 24-hour dietary recalls for the adult Swiss population who are French speaking [29,31,33,34]. It solicits information on consumption frequency and portion sizes during the 4 previous weeks for 97 fresh and prepared food items organized into 12 food groups (dietary supplements not included). Consumption frequencies range from “never during the last 4 weeks” to “2 or more times per day,” and portion sizes are recorded as equal to, or smaller or larger than, a reference size. The reference portions were defined as common household measures representing the median portion size of a previous validation study performed with 24-hour dietary recalls [29]. The “smaller” and “larger” portions represented the first and fourth quartiles of this distribution. We used the French Information Center on Food Quality (Maisons-Alfort Cedex, France) food-composition table to convert the food portions into macro- and micronutrients [35].

#### Urine Collection

CCSs received a home specimen collection kit including an information sheet on how to perform first morning urine spot collection, a 50 mL plastic specimen tube with a screw-on lid, a sealed plastic bag, and a bubble-lined return envelope with postage-paid labels addressed to the pediatric hematology-oncology unit of CHUV. We asked CCSs to collect a first morning urine sample, filling the tube up to 40 mL, and to seal the tube and write the sample date and time on the lid. We asked CCSs not to mark personal information on the tube to preserve confidentiality, and to send their sample by post. The medical staff of the pediatric hematology-oncology unit cooled the urine spot samples as soon as they received them. They divided the samples into one 8-mL aliquot for direct urine chemistry and nine 3-mL aliquots for biobank storage; the 8-mL sample was sent within 1 hour to the CHUV laboratory for analyses. Levels of potassium, sodium, phosphate, urate, urea, and creatinine were measured using routine laboratory procedures (Table 1). The 3-mL urine samples were frozen at –80°C and stored in a biobank at CHUV for later analyses.

**Table 1:** Primary and secondary end points and outcomes of interest.

End points and outcomes		Method	Quality promotion	(Expected) time point or window
**Primary**				
	Detailed dietary intake, macro- and micronutrients	Dietary intake assessed by a validated FFQ^a^ providing information on consumption frequency and portion sizes during the 4 previous weeks for 97 fresh and prepared food items organized in 12 food groups.	Validated FFQ	CCSs^b^ were expected to fill in and return the FFQ within 8 weeks. In case of nonresponse, a first and second reminder were sent.
	Urinary measurements	Laboratory methods: Sodium: indirect potentiometry Potassium: indirect potentiometry Urea: urease Urate: uricase Creatinine: Jaffe reaction Phosphate: phosphomolybdate	Standard laboratory procedures	Analyses were performed together with routine analyses in the hospital laboratory of Centre Hospitalier Universitaire Vaudois with Cobas 8000 (Roche Diagnostics). Analyses were performed during the whole study period.
**Secondary**				
	General response rate	The SCCSS^c^ tracking system tracked the number of CCSs who did not respond or declined participation.	N/A^d^	Evaluation after finalizing the study.
	Costs	Recording of costs, eg, laboratory, mailing, printing, urine collection sample kits.	N/A	Midterm evaluation and after finalizing the study.
	Participants’ reactions	Recording CCSs’ reactions by telephone, emails, or letter.	N/A	Evaluation after finalizing the study.

^a^FFQ: food frequency questionnaire.

^b^CCS: childhood cancer survivor.

^c^SCCSS: Swiss Childhood Cancer Survivor Study.

^d^N/A: not applicable.

### Data Management

#### Coding

We gave each participant an 8-digit identification (ID) code number to maintain anonymity. We used these ID codes in lieu of patient names for all data and urine spot samples. Data labelled with participant ID codes are stored on encrypted devices or secured servers. All participant data and biological samples are strictly confidential, and disclosure to third parties is prohibited. The coding key is stored at the Institute of Social and Preventive Medicine (ISPM), University of Bern, Bern, Switzerland, and is only available to authorized personnel.

#### Storage

All biomedical material is archived for 10 years at CHUV. In case there is no intention for use or a participant withdraws consent, the respective biological material will be destroyed. FFQ answers and urine spot laboratory results will be archived on servers of the ISPM, Lausanne, Lausanne, Switzerland, and ISPM, Bern for at least 10 years. Timelines that record and archive outcomes are in line with Swiss regulation. All results will be archived at and analyzed by ISPM, Bern as a nested study of the SCCSS.

### Statistical Analyses

We will include all CCSs who provided reliable dietary intake information and were neither pregnant nor lactating during the survey for FFQ analyses. Table 1 indicates the primary and secondary end points and outcomes of interest of the SCCSS-Nutrition study. We will evaluate whether CCSs meet dietary recommendations for Germany, Austria, and Switzerland [36]. We will compare mean intake with the recommended intake or, when not available, the adequate intake. We will calculate mean intake based on age and sex recommendations weighted by the age and sex distribution of the study population. Nutritional goals will be set at 100, where the mean intake meets the recommended or adequate intake. Total energy intake will be calculated including calories from alcohol consumption. We will calculate correlation coefficients to examine the strength and direction of the associations between the FFQ and urinary spot measurements. To validate the agreement between the 2 dietary assessment tools, we will perform cross-classification analyses to investigate whether the 2 dietary assessment tools rank CCSs’ dietary intake similarly. We will calculate the proportion of CCSs correctly classified in the same or contiguous category or in the opposite category (misclassified). We will use Bland-Altman plots to assess the level of agreement between the FFQ and the urine spot samples at the CCS group level. We will plot the difference between the 2 measurements against the mean of the 2 measurements for each CCS. We will use Stata (version 14; StataCorp LLC) for all analyses.

### Ethics

The cantonal ethics committee Commission cantonal d’éthique de la Recherche sur l’être humain, Lausanne approved the SCCSS-Nutrition study in March 2016. In July 2017, the cantonal ethics committee Geneva Commission Cantonal d’éthique de la Recherche approved the study with an amendment (protocol of both approvals: 2016-00031). Ethical approval of the SCCR and the SCCSS questionnaires was granted by the Ethics Committee of the Canton of Bern (KEK-BE: 166/2014).

## Results

### Characteristics of Participants and Nonparticipants

Table 2 presents the sociodemographic and lifestyle characteristics of both CCSs who completed the FFQ and those who did not, and those who participated in the collection of urine spot samples. The most common cancer diagnoses among CCSs completing the FFQ were leukemia, lymphoma, and central nervous system tumors (Table 3). Median age at diagnosis was 10 years (interquartile range 4-14 years) and median time from diagnosis to survey was 26 years (interquartile range 20-32 years). Of the 902 FFQ participants, 99 (12.34%) experienced a relapse.

### Costs

The costs of this study have remained within budget (Table 4). Costs include material, shipment of FFQs and urine spot sample collection kits, reminders, data entry, data management, and laboratory urine analyses.

**Table 2:** Sociodemographic and lifestyle characteristics of participants and nonparticipants in the food frequency questionnaire (FFQ) and the urine spot sample collection.

Characteristics		FFQ		Urine spot sample	
		Participants (n=802)	Nonparticipants^a^ (n=797)	Participants (n=111)	Nonparticipants^b^ (n=110)
Male sex, n (%)		401 (50.0)	443 (55.6)	49 (44.1)	53 (48.2)
**Age at survey (years), n (%)**					
	≤30	248 (30.9)	328 (41.2)^c^	26 (23.4)	44 (40.0)
	31-39	320 (39.9)	305 (38.3)	37 (33.3)	37 (33.6)
	≥40	234 (29.2)	164 (20.6)	48 (43.2)	29 (26.4)
**Country of birth, n (%)**					
	Switzerland	763 (95.1)	736 (92.3)	101 (91.0)	98 (89.1)
	Other	39 (4.9)	60 (7.5)	10 (9.0)	12 (10.9)
	Missing data	N/A^d^	1 (0.1)	N/A	N/A
**Education (highest degree), n (%)**					
	Lower than university	527 (65.7)	681 (85.4)^e^	69 (62.2)	71 (64.6)
	University	270 (33.7)	98 (12.3)	42 (37.8)	39 (35.5)
	Missing data	5 (0.6)	18 (2.3)	N/A	N/A
**Living situation, n (%)**					
	Alone	164 (20.4)	129 (16.2)^e^	19 (17.1)	24 (21.8)
	Other	634 (79.1)	655 (82.1)	91 (82.0)	86 (78.2)
	Missing data	4 (0.5)	13 (1.6)	1 (0.9)	N/A
**Physical activity^f^, n (%)**					
	Inactive	165 (20.6)	204 (25.6)^e^	32 (28.8)	32 (29.1)
	Active	628 (78.3)	572 (71.8)	76 (68.5)	77 (70.0)
	Missing data	9 (1.1)	21 (2.6)	3 (2.7)	1 (0.9)
**Smoking status, n (%)**					
	Never	532 (66.3)	511 (64.1)^e^	69 (62.2)	65 (59.1)
	Former	132 (16.5)	79 (9.9)	15 (13.5)	24 (21.8)
	Current	128 (16.0)	207 (26.0)	25 (22.5)	21 (19.1)
	Missing data	10 (1.3)	N/A	2 (1.8)	N/A
**Body mass index at survey (kg/m^2^), n (%)**					
	Underweight (<18.5)	39 (4.9)	57 (7.2)^e^	7 (6.3)	3 (2.7)
	Normal (18.5-24.9)	490 (61.1)	500 (62.7)	76 (68.5)	67 (60.9)
	Overweight (25-29.9)	177 (22.1)	141 (17.7)	15 (13.5)	25 (22.7)
	Obese (≥30)	75 (9.4)	59 (7.4)	10 (9.0)	14 (12.7)
	Missing data	21 (2.6)	40 (5.0)	3 (2.7)	1 (0.9)

^a^Includes 635 childhood cancer survivors (CCSs) who did not respond, 45 who declined, 11 who were pregnant or breastfeeding, 35 with missing dietary data, and 71 with unreliable dietary data.

^b^Includes 9 CCSs with no valid address available anymore or who were abroad, 15 who declined, and 86 who did not respond.

^c^Age at survey is calculated for FFQ nonparticipants by taking the average participants’ date of filling in the questionnaire.

^d^N/A: not applicable.

^e^Based on information from the Swiss Childhood Cancer Survivor Study baseline questionnaire filled in between 2007 and 2013 by FFQ nonparticipants.

^f^Active: ≥150 minutes of moderately intense or 75 minutes of vigorously intense or a combination of moderately and vigorously intense physical activity per week.

**Table 3:** Clinical characteristics of participants and nonparticipants in the food frequency questionnaire (FFQ) and the urine spot sample collection.

Characteristics		FFQ		Urine spot sample	
		Participants (n=802)	Nonparticipants^a^ (n=797)	Participants (n=111)	Nonparticipants^b^ (n=110)
**ICCC-3^c^ diagnosis, n (%)**					
	I: Leukemia	246 (30.7)	264 (33.1)	30 (27.0)	27 (24.6)
	II: Lymphoma	173 (21.6)	139 (17.4)	30 (27.0)	30 (27.3)
	III: CNS^d^ tumor	81 (10.1)	140 (17.6)	9 (8.1)	17 (15.5)
	IV: Neuroblastoma	28 (3.5)	31 (3.9)	4 (3.6)	3 (2.7)
	V: Retinoblastoma	12 (1.5)	22 (2.8)	2 (1.8)	3 (2.7)
	VI: Renal tumor	52 (6.5)	41 (5.1)	4 (3.6)	3 (2.7)
	VII: Hepatic tumor	6 (0.8)	3 (0.4)	1 (0.9)	1 (0.9)
	VIII: Bone tumor	50 (6.2)	29 (3.6)	11 (9.9)	5 (4.6)
	IX: Soft tissue sarcoma	66 (8.2)	32 (4.0)	7 (6.3)	10 (9.1)
	X: Germ cell tumor	43 (5.4)	42 (5.3)	8 (7.2)	4 (3.6)
	XI and XII: Other tumor	26 (3.2)	17 (2.1)	4 (3.6)	3 (2.7)
	Langerhans cell histiocytosis	19 (2.4)	37 (4.6)	1 (0.9)	4 (3.6)
**Age at diagnosis (years), n (%)**					
	<5	251 (31.3)	262 (32.9)	28 (25.2)	29 (26.4)
	5-9	164 (20.4)	211 (26.5)	19 (17.1)	23 (20.9)
	10-14	239 (29.8)	222 (27.9)	34 (30.6)	27 (24.6)
	15-20	148 (18.5)	102 (12.8)	30 (27.0)	31 (28.2)
Time since diagnosis (years), median (interquartile range)		26.1 (20.2-31.7)	N/A^e^	28.3 (21.0-32.7)	22.8 (18.5-30.1)
History of relapse, n (%)		99 (12.3)	107 (13.4)	18 (16.2)	15 (13.6)

^a^Includes 635 childhood cancer survivors (CCSs) who did not respond, 45 who declined, 11 who were pregnant or breastfeeding, 35 with missing dietary data, and 71 with unreliable dietary data.

^b^Includes 9 CCSs with no valid address available anymore or who were abroad, 15 who declined, and 86 who did not respond.

^c^ICCC3: International Childhood Cancer Classification, Third Edition.

^d^CNS: central nervous system.

^e^N/A: not applicable.

**Table 4:** Costs to perform the Swiss Childhood Cancer Survivor Study-Nutrition study.

Expenses	Costs (US $)
Material, eg, (return) envelopes, questionnaires, urine tubes	6514
Address update for childhood cancer survivors	20,232
Mailings	7125
Data entry for food frequency questionnaires	13,360
Laboratory analyses of urine spot samples	2908
Ethics committee approval	602
Total costs	50,741

### Childhood Cancer Survivor Reactions

CCSs had varied reactions to the FFQ. The majority of CCSs wanted to participate and welcomed a follow-up questionnaire. Only a small number of the 1599 CCSs to whom FFQs were sent (n=45, 2.81%) declined to complete the FFQ. Of the 221 CCSs to whom information letters for urine collection were sent, 15 declined to collect a urine spot sample (Figure 1). All CCSs who declined participation expressed a willingness to participate in future studies. A total of 26 CCSs contacted us by telephone (n=13), email (n=5), or letter (n=8) with questions about the purpose of the study, its setup, eligibility, or another question, or to notify the study team about a delay in FFQ response or urine collection. Overall, the CCSs were supportive and open to participation in the study. We received no angry or aggressive reactions.

We anticipate that the results of the SCCSS-Nutrition study will be available mid-2019.

## Discussion

### Principal Findings

SCCSS-Nutrition is, to our knowledge, the first study in Switzerland that has collected in-depth dietary data. It will allow researchers to assess dietary intake and quality in CCSs and to compare 2 dietary assessment tools: urine measurements and FFQs. Urine spot sample measurements can quantify nutrient intake objectively and can therefore complement self-reported dietary information from the FFQ.

Unhealthy dietary intake is an important element in the development of chronic morbidities such as type 2 diabetes, metabolic syndrome, and CVD in the general population. Populations with these morbidities are therefore widely recommended to consume a healthy and balanced diet. The extensively investigated Mediterranean diet, with high intakes of fish, fruit, vegetables, legumes, nuts, whole grains, and monounsaturated fats from olive oil, has been shown to reduce, or even prevent, CVD, diabetes, obesity, metabolic syndrome, and cancer in the general population [37-41] and in CCSs [5]. This makes nutrition one of the main determinants of health in the general population, and is particularly relevant for people with additional risk factors, including CCSs. Nevertheless, knowledge about CCSs’ dietary intake and their nutritional status is lacking within Switzerland and is limited worldwide.

### Strengths and Limitations

This study, nested within the SCCSS, assesses dietary intake information of CCSs and compares 2 dietary assessment tools: the FFQ and dietary measurements from urine spot samples. We found the SCCSS-Nutrition study to be well received and feasible. This is, to our knowledge, the first study to provide detailed dietary information on Swiss CCSs and to demonstrate the feasibility of such a study. With the addition of dietary indicators from urine spot samples, SCCSS-Nutrition makes further comparison possible. Additionally, we had high response rates for completing the FFQ and collecting urine spot samples. Finally, we have access to detailed sociodemographic data from the SCCSS baseline and follow-up questionnaire, and high-quality clinical information extracted from medical records in the SCCR. This a very rich dataset available for analysis.

Limitations of this study were that some CCSs said the FFQ was too long. This might have influenced CCSs to either under- or overreport dietary intake. Also, we asked CCSs for a single spot urine sample rather than multiple spot samples or a 24-hour urine collection to minimize participation burden. Comparison of the self-reported FFQ data, representing habitual dietary intake over 4 weeks, with urine spot analysis data, indicative of the dietary intake during the day before, should therefore be regarded with caution. Seasonal influences could play a role in the FFQ assessment, as we assessed dietary intake for the past 4 weeks rather than the past year. Finally, the interval between the FFQ assessment and urine spot collection could produce differences in dietary intake due to seasonal influences.

### Lessons Learned

Setting up this study provided valuable insight into several methodological and logistic issues. We asked CCSs to return urine samples within 2 weeks and to post their urine samples between Monday and Thursday. This prevented the samples from arriving during the weekend. The time frame of 2 weeks was too short; several CCSs contacted us to ask for an extension. The urine collection tubes had a diameter of 3 cm and did not fit the opening slit of an official Swiss mailbox when the CCSs placed a sample in a sealed plastic bag and a bubble-lined postal return envelope. Given this, the response rate was higher than we expected, and we reached the recruitment target because of the up-to-date address list and personal information of SCCR, and the high motivation of CCSs to participate. Furthermore, including a study center took longer than expected, due to arranging appropriate urine storage within the hospital, and an extra briefing about the potential hazards of CCSs’ urine contaminated with chemotherapeutic agents in case of cancer recurrence to safeguard the safety of laboratory staff.

### Conclusions

The SCCSS-Nutrition study collected in-depth dietary data that will enable an assessment of dietary intake and dietary quality in CCSs and a comparison of dietary assessment tools. The study will help fill nutrition knowledge gaps and is a first step toward surveillance guidelines and targeted nutritional recommendations in Switzerland.

## Appendix

Multimedia Appendix 1 Food frequency questionnaire in French.

Multimedia Appendix 2 Peer-review report ethical committee.

## Abbreviations

CCS: childhood cancer survivor
CHUV: Centre Hospitalier Universitaire Vaudois
CVD: cardiovascular disease
FFQ: food frequency questionnaire
ID: identification
ISPM: Institute of Social and Preventive Medicine
SCCR: Swiss Childhood Cancer Registry
SCCSS: Swiss Childhood Cancer Survivor Study
SCCSS-Nutrition: Swiss Childhood Cancer Survivor Study-Nutrition study
