# Supplementary material for: Nutritional Assessment of Childhood Cancer Survivors (the Swiss Childhood Cancer Survivor Study-Nutrition): Protocol for a Multicenter Observational Study
Source: JMIR Res Protoc. 2019 Nov 18;8(11):e14427. doi: 10.2196/14427 (PMC6887820; doi:10.2196/14427)
Supplement: Multimedia Appendix 1 [file resprot_v8i11e14427_app1.pdf]

Appendix 1. Food frequency questionnaire in French

ALIMENTATION

1. Suivez-vous un régime alimentaire?

Non ☐
Oui ☐

Si oui, lequel?

2. Lequel des aliments suivants **excluez-vous totalement** de votre alimentation? ***(plusieurs choix possible)***

œufs ☐
viande (bœuf, porc, veau, etc.) ☐
volaille (poulet, dinde, canard etc.) ☐
fruits ☐
légumes ☐
poissons ☐
fruits de mer ☐
fromages ☐
lait ☐
autres produits laitiers ☐
fruits à coque ☐
céréales ☐
alcool ☐
aucun des aliments ci-dessus ☐

Instructions pour remplir le questionnaire

Par ce questionnaire, nous souhaitons connaître votre alimentation au cours des **4 dernières semaines.**

Pour chaque type d'aliment, nous vous demandons de **comparer votre portion habituelle à la portion de référence**, selon l'exemple ci-dessous.

Exemple
Cette personne a mangé le mois dernier du pain complet midi et soir, donc 2 fois par jour; elle a mangé 2 tranches de pain à chaque fois, donc plus que la portion de référence.
Elle a mangé du beefsteak 1 à 2 fois par semaine, sa tranche de viande pesant environ 150 grammes donc pareille à la portion moyenne de référence.

ALIMENTS	FREQUENCE							QUANTITE			
	Jamais ces 4 dernières semaines	1 x par mois	2 à 3 x par mois	1 à 2 x par semaine	3 à 4 x par semaine	1 x par jour	2 x ou plus par jour	Portion de référence	Votre portion		
									moins	pareil	plus
Pain complet, au seigle							X	50g = 1 tranche			X
Beefsteak, cheval, veau (escalope, filet)				X				150g		X	

Avec les questions suivantes, nous aimerions savoir votre alimentation au cours **des 4 dernières semaines.**

ALIMENTS		FREQUENCE							QUANTITE			
		Jamais ces 4 dernières semaines	1 x par mois	2 à 3 x par mois	1 à 2 x par semaine	3 à 4 x par semaine	1 x par jour	2 x ou plus par jour	Portion de référence	Votre portion		
										moins	pareil	plus
**Fromages, Yaourts** 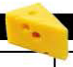												
1	yaourt nature								1 yaourt = 180g			
2	yaourt light, flan light, crème light								1 yaourt = 180g			
3	yaourt aux fruits, flan, crème dessert								1 yaourt = 180g			
4	fromage blanc 0%								120g			
5	fromage blanc 20%, ricotta, séré, cottage-cheese								100g			
6	feta, mozzarella, fromage frais ½ sel, petit-suisse								40g = 1 petit-suisse			
7	gruyère, tomme, camembert, bleu, parmesan								40g = taille d'une boîte d'allumettes			
8	fondue au fromage								20 bouchées = 3 à 4 tranches de pain			
**Pain, Céréales** 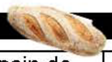												
9	pain blanc, pain de mie, de campagne, au lait, tresse								50g = 1 tranche			
10	pain complet, pain au seigle								50g = 1 tranche			
11	müesli ou autres céréales mélangées								50g = ½ gobelet de yaourt			
12	corn-flakes, blé soufflé, céréales soufflées								30g = 1 gobelet de yaourt			
13	biscottes, cracottes, pain grillé «suédois»								18g = 3 pièces			

ALIMENTS		FREQUENCE							QUANTITE			
		Jamais ces 4 dernières semaines	1 x par mois	2 à 3 x par mois	1 à 2 x par semaine	3 à 4 x par semaine	1 x par jour	2 x ou plus par jour	Portion de référence	Votre portion		
										moins	pareil	plus
**Viandes** 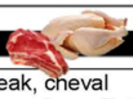												
14	beefsteak, cheval veau (escalope, filet)								150g			
15	poulet sans peau								80g = 1 blanc de poulet			
16	poulet avec peau								80g = 1 blanc de poulet			
17	steak haché, entrecôte, rôti (bœuf, porc, veau)								100g			
18	jambon, pot-au-feu, côtelettes d'agneau, côtes de porc								50g = 2 tranches fines de jambon ou 1 côtelette			
19	saucisson, jambon cru, lard maigre, salami								50g = 2 tranches			
20	pâté, terrine								50g = ½ tranche			
21	cervelas								1 pièce			
22	saucisse								1 pièce			
23	foie de veau, de génisse, de porc								130g = 1 tranche			
24	foie de volaille								50g = 1 pièce			
**Poissons, Crustacés** 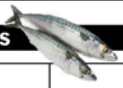												
25	saumon frais, saumon fumé								100g = 1 darne = 2 tranches			
26	poisson frit, pané, filets de perche								150g = 3 grand sticks			
27	thon à l'huile								40g = ½ boîte			
28	poisson maigre type cabillaud, colin, truite								150g			
29	crevettes, coquillages								80g			

ALIMENTS		FREQUENCE							QUANTITE			
		Jamais ces 4 dernières semaines	1 x par mois	2 à 3 x par mois	1 à 2 x par semaine	3 à 4 x par semaine	1 x par jour	2 x ou plus par jour	Portion de référence	Votre portion moins	pareil	plus
**Légumes** 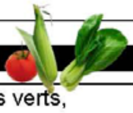												
30	haricots verts, épinards								180g = 1 gobelet yaourt			
31	choux-fleurs, brocolis								180g = 1 gobelet yaourt			
32	tomates								180g = 1 grosse ou 2 petites tomates			
33	carottes								100g = 1 gobelet yaourt			
34	salade verte								100g = 1 gobelet yaourt			
35	vinaigrette								15g = 1 cuill. à soupe			
36	petits pois, maïs en grains								75g = ½ gobelet yaourt			
37	avocat								100g = ½ avocat			
38	soupe de légumes claire (bouillon de légumes)								300ml= 1 bol ou 1 assiette à soupe			
39	soupe de légumes épaisse (aux pois, aux haricots, minestrone)								300ml= 1 bol ou 1 assiette à soupe			
40	pommes de terre nature								150g= 1 grosse ou 2 petites p. de terre			
41	frites								120g = 1 poignée			

	ALIMENTS	FREQUENCE							QUANTITE			
		Jamais ces 4 dernières semaines	1 x par mois	2 à 3 x par mois	1 à 2 x par semaine	3 à 4 x par semaine	1 x par jour	2 x ou plus par jour	Portion de référence	Votre portion		
										moins	pareil	plus
**Autres**												
42	pâtes alimentaires								180g cuites = 70g crues			
43	raviolis, tortellinis, cannellonis								150g cuits = env. 10 raviolis			
44	riz								150g cuit = 60g cru			
45	sauce tomate								80g = 2 cuill. à soupe			
46	semoule de blé, couscous								80g cuit = 2 cuill. à soupe			
47	pizza								1/2 pizza de la pizzeria			
48	quiche lorraine								100 g = 1 ramequin			
49	œufs								2			
50	tofu								65g			
**Vous ajoutez sur vos plats ou sur votre pain?** 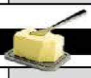												
51	margarine allégée								10 g = 1 cuill. à café			
52	beurre								10g = portion bistrot			
53	crème fraîche entière 35%								20g= 1 cuill. soupe			
54	mayonnaise								10 g = 1 cuill. à café			
**Fruits** 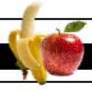												
55	banane, pomme, poire, prune, raisin								150g = 1 fruit ou 1 petite grappe			
56	orange, mandarine, clémentine								150g = taille d'une orange			
57	pêche, nectarine, abricot, melon								150g = taille d'une pêche			
58	fraises, framboises, myrtilles, cassis								120g = 1 petite barquette			
59	kiwi								80g = 1 fruit			
60	fruits en conserve, compote sucrée								150g = taille du gobelet de yaourt			

	ALIMENTS	FREQUENCE							QUANTITE			
		Jamais ces 4 dernières semaines	1 x par mois	2 à 3 x par mois	1 à 2 x par semaine	3 à 4 x par semaine	1 x par jour	2 x ou plus par jour	Portion de référence	Votre portion moins	Votre portion pareil	Votre portion plus
	**Pâtisseries, Sucreries**											
61	croissant, pain au chocolat								1 pièce			
62	tarte aux fruits								120g = 1 tranche			
63	gâteau à la crème								90g = 1 gâteau			
64	cake, pâtisserie sèche								90g= 1 grosse tranche			
65	biscuits secs, cookies								20g= 3 biscuits			
66	chocolat								15g= 3 carrés			
67	confiture, miel								20g = 1 cuill. à soupe			
68	glace, sorbet								100g = 1 boule			
69	sucre artificiel (assugrin, aspartam)								1 comprimé			
70	sucre								1 morceau et ½			
	**Pour cuisiner**											
71	beurre								10g = portion bistrot			
72	margarine								10g = 1 cuill. à café			
73	huile d'olive								2 cuill. à café			
74	huile d'arachide								2 cuill. à café			
75	huile de tournesol								2 cuill. à café			
	**Vitamines, suppléments alimentaires**											
76	vitamine C								500 mg			
77	vitamine E								1'000 mg			
78	multivitamines								1 comprimé			
79	son								1 cuill. à soupe			
80	comprimé d'ail								1 comprimé			

ALIMENTS		FREQUENCE							QUANTITE			
		Jamais ces 4 dernières semaines	1 x par mois	2 à 3 x par mois	1 à 2 x par semaine	3 à 4 x par semaine	1 x par jour	2 x ou plus par jour	Portion de référence	Votre portion moins	pareil	plus
**Boissons**												
81	café								1 tasse			
82	lait écrémé (à 0%) dans le café								2 cuill. à soupe			
83	lait entier ou mi-écrémé dans le café								2 cuill. à soupe			
84	crème à café								1 portion de bistrot			
85	lait écrémé (à 0%) à boire								2dl = 1 verre			
86	lait entier ou mi-écrémé à boire								2dl = 1 verre			
87	Aproz, Valser, San Pellegrino, Passuger								2dl = 1 verre			
88	Perrier, Vittel, Volvic								2dl = 1 verre			
89	eau du robinet, Henniez, Evian, Vichy								2dl = 1 verre			
90	limonade, coca, soda, sirop de fruits								2dl = 1 verre			
91	jus de fruits frais								2dl = 1 verre			
92	jus de fruits en bouteille ou en brique								2dl = 1 verre			
93	thé, infusion								2dl = 1 tasse			
94	bière								3dl = 1 cannette			
95	vin, champagne								1dl et ½ = 1 verre à vin			
96	apéritifs type anisette, Martini								½ dl = ¼ de verre			
97	alcool fort type whisky, eau de vie, liqueur								1dl = ½ verre			

ALIMENTS		FREQUENCE							QUANTITE			
		Jamais ces 4 dernières semaines	1 x par mois	2 à 3 x par mois	1 à 2 x par semaine	3 à 4 x par semaine	1 x par jour	2 x ou plus par jour	Portion de référence	Votre portion		
										moins	pareil	plus
**Autres**												
98	oignons								1 pièce			
99	concombres, courgettes, aubergines								150g = 1 gobelet yaourt			
100	poivrons								½ pièce			
101	champignons								150g = 1 gobelet yaourt			
102	autres huiles, par ex. huile de colza								2 cuill. à café			
103	sauces grasses, par ex. sauce à la crème, sauce avec du lait de coco								½ dl = 3 cuill. à soupe			
104	sauce bolognaise								160g = 4 cuill. à soupe			
105	fruits à coque (amandes, noix, cacahuètes, noisettes etc.)								1 poignée			
106	sel ajouté								1 pincée			
107	chips								1 poignée			
108	olives noires et vertes								5 pièces			
109	autres fromages, (Emmental, fromage à raclette etc.)								40g = taille d'une boîte d'allumettes			
110	chocolat chaud ou froid, boisson mélangée à base de lait, shake de protéines								2dl = 1 verre			
111	Nutella								20g = 1 cuill. à soupe			
112	birchermüesli avec yaourt								180g = 1 gobelet yaourt			

ALIMENTS	FREQUENCE							QUANTITE			
	Jamais ces 4 dernières semaines	1 x par mois	2 à 3 x par mois	1 à 2 x par semaine	3 à 4 x par semaine	1 x par jour	2 x ou plus par jour	Portion de référence	Votre portion		
									moins	pareil	plus

S'il manque des aliments que vous avez mangés le mois dernier, décrivez-les-ci-dessous

113											
114											
115											
116											
117											
118											

Sur cette page vous trouvez de la place pour partager avec nous vos questions, remarques, critiques et suggestions à propos de l'étude. Nous vous sommes très reconnaissants pour vos commentaires.
