# Supplementary material for: Nutritional Assessment of Childhood Cancer Survivors (the Swiss Childhood Cancer Survivor Study-Nutrition): Protocol for a Multicenter Observational Study
Source: JMIR Res Protoc. 2019 Nov 18;8(11):e14427. doi: 10.2196/14427 (PMC6887820; doi:10.2196/14427)
Supplement: Multimedia Appendix 2 [file resprot_v8i11e14427_app2.pdf]

COMMISSION CANTONALE
D'ÉTHIQUE DE LA RECHERCHE
SUR L'ÊTRE HUMAIN
CER-VD
Av. de Chailly 23
1012 Lausanne

Dr. Med. Maja Beck Popovic
CHUV
Rue du Bugnon 46
1011 Lausanne

Lausanne, le 3 février 2016
Réf. AP/sm/cc

Décision de la Commission cantonale (VD) d'éthique de la recherche sur l'être humain (CER-VD)

No de protocole	2016-00031
Titre	SCCSS Pilot study: urine spot collection among childhood cancer survivors - **Thèse de doctorat de M. Fabien Belle**
Investigateur principal	Dr. Med. Maja Beck Popovic
Date de soumission	11.01.2016

I. Procédure

La CER-VD a statué en :

Procédure ordinaire	☐	
Procédure simplifiée	☑	26.01.2016
Décision présidentielle	☐	

II. Décision

La décision concerne: VD CHUV

☐ **Autorisation accordée**
☐ **Autorisation avec charges**

P:\CER\PROTOCOLES 2016\Décisions\2016-00031 160203 PS DEC C.docx

☑ **En l'état, l'autorisation ne peut pas être accordée**

☐ **Autorisation non accordée**

☐ **Non entrée en matière**

Signification: L'étude ne peut pas commencer.
Prière de répondre point par point aux conditions de la CER-VD et de nous faire parvenir les documents révisés avec les modifications apparentes et la mention de la date de la nouvelle version.

Les Conditions sont en gras, les charges en écriture normale, *les commentaires/suggestions en italique.*

Considération générale :

1. Selon la documentation soumise, l'envoi du questionnaire précède celui du formulaire d'information et de consentement. Si c'est bien le cas, la CER-VD vous prie de lui expliquer les raisons de ce choix car il semble plus rationnel de collecter d'abord le consentement puis de faire remplir les questionnaires uniquement aux participant(e)s qui ont donné leur consentement pour la participation à l'étude avec le don d'urine.

2. Comment les "outpatients" seront-ils recrutés ? Le cas échéant, veuillez nous soumettre une copie du courrier qui leur sera adressé. De plus, le fait de recontacter à plusieurs reprises ces patient(e)s dans un court laps de temps ne risque-t-il pas de raviver des souvenirs douloureux ou du moins de les irriter? Il est aussi imaginable que pour "avoir la paix" certains patients répondent aux questionnaires de façon approximative. Dans le but de diminuer ce risque la CER vous suggère de prévoir la possibilité pour les personnes contactées de retourner un document indiquant qu'elles ne souhaitent pas participer et qu'elles ne souhaitent plus être contactées.

3. Dans quelle mesure ne serait-il pas envisageable de demander aux "outpatients" acceptant de participer à l'étude de se rendre à l'hôpital pour la présentation de l'étude et des questionnaires et la collecte de l'urine afin d'assurer une qualité uniforme des données et des échantillons collectés

Protocole.

4. Page 4, second endpoint : comment est évalué le "response rate" pour les "outpatients" : par rapport au premier envoi du FFQ ou par rapport à l'envoi du formulaire d'information et de consentement?

5 Section 2.1: Swismedic n'est pas impliqué dans l'approbation des études de ce type et il n'est pas prévu de contrôles dans ce type d'étude par des autorités. A modifier dans le protocole et la feuille d'information.

6. Veuillez spécifier le devenir des données collectées après la fin de l'étude et dans le cas où le participant retire son consentement.

7. Veuillez mentionner le risque lié à la protection des données (risque de diffusion d'information médicale)

8. Section 6.1 : y a-t-il un âge maximum pour l'inclusion dans l'étude ?

Formulaire d'information et de consentement :

9. Voir les commentaires dans le document annexé.

10. Veuillez spécifier dans la feuille d'information que la participation n'engendra aucun coût pour l'expédition des échantillons d'urine (outpatients).

Règlement biobanque :

11. Il s'agit du règlement de la biobanque sur la Pression artérielle et rein. Quel est le lien avec l'étude ?

Questionnaire :

12. Remplacer la date de naissance par le code.

CV Investigateur principal :

13. *Daté de Novembre 2014, et non du 14.12.2015 comme indiqué dans le formulaire de soumission électronique. Dans le formulaire de soumission électronique, la date saisie dans le champ "Date of doc." doit être celle du document lui-même et non celle de sa saisie dans le formulaire.*

III. Classification

☑ **Projet de recherche au sens de l'ORH:**

recherche sur des personnes	Catégorie ☑ A	☐ B
réutilisation du matériel biologique ou des données personnelles liées à la santé		☐
personnes décédées		☐
embryons et des fœtus		☐
avec rayonnements ionisants		☐

IV. Justifications de la décision/Remarques

Pas de remarque

V. Taxes et émoluments

Code tarifaire 5.0 Taxe CHF 600.-

VI. Voies de recours

La présente décision peut faire l'objet d'un recours au Tribunal cantonal, Cour de droit administratif et public. L'acte de recours doit être déposé auprès du Tribunal cantonal dans les **30 jours** suivant la communication de la décision attaquée ; il doit être signé et indiquer les conclusions et motifs du recours. La décision attaquée est jointe au recours. Le cas échéant, ce dernier est accompagné de la procuration du mandataire.

VII. Communication au requérant, et en plus à:

Promoteur	☐	Swissmedic	☐	OFSP	☐
Autres	☑	Fabiën Belle-van Sprunde, PhD student, fabien.belle@unil.ch			

VIII. Composition de la Commission lors de la prise de décision (en annexe)

Prof. André Pannatier
Vice-président

Annexes : Composition de la Commission lors de la séance du 26.01.2016
Feuilles d'information et de consentement annotées par la CER-VD

La décision de la CER-VD se base sur les documents soumis via le portail électronique. Selon vos indications il s'agit de:

1. Cover Letter
0_SCCSS_lettre accompagnement_version1_14.12.2015.docx 14/12/2015
2. Synopsis of the study plan
see doc/cat: 4, page/ref: 4-5
3. Participant information sheet and informed consent (ICF)
3_SCCSS_Participant information sheet_version1_14.12.2015.docx 14/12/2015 1
4. Study plan (protocol), signed and dated
4_SCCSS_HRO_Research_Plan_version1_14.12.2015.docx 14/12/2015 1
6. Investigator's CV, dated
6_CV Dr. Med. Maja Beck Popovic.pdf 14/12/2015
6_CV Prof. Dr. Med. Murielle Bochud.pdf 14/12/2015
6_CV Prof. Dr. Med. Claudia Kuehni.pdf 14/12/2015
8. Details on infrastructure suitability and availability at the location where the trial is executed
8_SCCSS_Disponibilité des infrastructures et adéquation_version1_14.12.2015.docx 14/12/2015
11. Other documents handed over to study participants
11_SCCSS_Urine collection instructions_version1_14.12.2015.docx 14/12/2015 1
11_SCCSS 14/12/2015 1
12. Details on nature and scope/value of compensation for participants
There is no compensation for the participation in this study
13. Other personnel
13_SCCSS_Other personnel_version1_14.12.2015.docx 14/12/2015
14. Information on secure handling of biological material and personal data, and in particular on the storage thereof
see doc/cat: 4, page/ref: p9 (2.5), p14 (5.2), p19 (9.3), p20 (9.4)
39. Miscellaneous / Varia
SKIPOGH2_V1_Reglement_biobanque_12.07.121.doc 22/05/2012 1

La CER-VD s'aligne sur les principes ICH GCP

Obligations du requérant (promoteur ou investigateur):
1. En cas de révision, les documents ainsi que la liste de vérification actualisée sont envoyés à la CER-VD sous forme papier et CD-Rom. La liste de vérification ne répertorie que les documents révisés;
2. Les événements indésirables graves, la fin ou l'arrêt prématuré d'un essai clinique et les modifications essentielles sont annoncés selon les dispositions légales en vigueur.
3. Le rapport final est envoyé à la CER-VD dans un délai d'un an au plus tard.
4. Les essais cliniques sont enregistrés dans un registre primaire de l'OMS (WHO-Primärregister puis dans la banque de données complémentaire de la Confédération (Swiss National Clinical Trials Portal [SNCTP];
5. Pro memoria: Démarche pour la soumission des documents révisés:
- Les documents révisés et la liste de vérification actualisée sont mis à disposition des commissions d'éthique sous forme digitale, et/ou d'un exemplaire papier.
- La liste de vérifications répertorie uniquement les documents révisés.
- Les modifications doivent être signalées dans les documents révisés.
- Les documents révisés sont mis à disposition des autorités compétentes pour approbation.

Composition de la Commission cantonale (VD) d'éthique de la recherche sur l'être humain lors de la séance du 26 janvier 2016

Procédure simplifiée

L'avis de la Commission d'Ethique ayant siégé dans sa composition détaillée ci-après est valable, le quorum étant atteint (art. 6 de l'Ordonnance d'organisation concernant la LRH du 20 septembre 2013).

					participe à l'avis	
	**Nom, prénom**	**Profession, titre**	H	F	**oui**	**non**
**Présidence**	Pannatier André	Vice-président, Professeur en pharmacie	☒	☐	☒	☐
**Membres**	Bidiville Jacques	Spécialiste en médecine interne	☒	☐	☒	☐
	Martinuz Marco	Administrateur	☒	☐	☒	☐

Mmes Chantal Camani Gavin et Coralie Hutchison, Adjointes scientifiques, ont assisté à la séance.

canton de vaud
LIBERTÉ ET PATRIE
COMMISSION CANTONALE
D'ÉTHIQUE DE LA RECHERCHE
SUR L'ÊTRE HUMAIN
CER-VD
Av. de Chailly 23
1012 Lausanne

Pour la facturation

**Titre**	SCCSS Pilot study: urine spot collection among childhood cancer survivors - **Thèse de doctorat de M. Fabien Belle**
**N° du protocole**	2016-00031
**Adresse de facturation**	Prof. Dr. Med. Murielle Bochud<br>Route de la Corniche 10<br>1010 Lausanne
**Montant**	Code tarifaire 5.0 Taxe CHF 600.-
**N° de la facture**	N° 2016-00031030216

La facture sera envoyée ultérieurement.

Lausanne, le 3 février 2016

Prof. Patrick Francioli
Président

IUMSP - Institut universitaire de médecine sociale et préventive
Biopôle 2
Route de la Corniche 10
CH-1010 Lausanne

Etude suisse sur la
Santé des personnes atteintes d'un cancer, d'une tumeur ou d'une leucémie durant l'enfance

**Information courte**	Voir détails page:
**<u>SCCSS Etude pilote: collecte d'urine fraîche parmi les survivants de cancer infantile</u>**<br>(SCCSS Etude pilote : collecte d'urine fraîche parmi des survivants de cancer infantile dans un centre unique en vue d'évaluer la consommation nutritionnelle)	3
**Les raisons pour lesquelles nous nous adressons à vous:**<br>Nous travaillons actuellement sur la consommation alimentaire des survivants de cancer infantile. Comme vous avez justement souffert d'un cancer pendant votre enfance, nous souhaiterions vous ~~demander~~ proposer de participer à notre projet de recherche. C'est la raison pour laquelle nous vous faisons parvenir cette feuille d'informations.	3
**Les objectifs que nous voulons atteindre avec notre étude:**<br>Cette étude a pour but de comparer un questionnaire sur votre consommation alimentaire avec la présence de certaines molécules dans un échantillon de votre urine. De plus, nous souhaiterions investiguer sur la faisabilité de cette étude. Au vu de ces expériences, nous espérons ainsi planifier une étude qui sera menée dans toute la Suisse.	3
**Ce que votre participation à l'étude signifie pour vous:**<br>Vous allez recevoir prochainement une lettre de votre médecin personnel, par courrier ou en main propre, vous demandant votre accord de participation. En cas d'accord de votre part et après signature d'une attestation de consentement éclairé, vous recevrez un kit de collecte d'urine, et un questionnaire sur votre consommation alimentaire. Nous vous demanderons de collecter un échantillon de première urine matinale à votre domicile ou à l'hôpital. Après avoir collecté votre urine, vous pourrez soit l'envoyer par courrier ou la remettre à votre médecin. La collecte d'urine ne devrait pas vous prendre plus de 5 minutes.	3/4
**Les bénéfices et les risques que l'étude représente pour vous:**<br>Ce projet évaluera votre consommation alimentaire. Nous souhaiterions ainsi examiner les associations potentielles entre la consommation alimentaire et la santé. Par ailleurs, nous aimerions explorer les besoins de recommandations pour une alimentation spécifique concernant les survivants de cancer infantile. Cette étude ne présente aucun risque pour vous.	4
**Vos droits si vous participez à l'étude:**<br>Vous êtes libre d'accepter ou de refuser de participer à l'étude. Si vous décidez de ne pas participer, cela ne changera rien à votre prise en charge médicale. Si vous décidez de participer, vous pourrez à tout moment revenir sur votre décision et vous retirer de l'étude. Vous n'avez pas à justifier votre décision.<br>Pendant l'étude, nous serons amenés à recueillir des données médicales vous concernant. Nous devrons en outre collecter un échantillon de votre urine. Si vous	4/5

Commentaire [CER1]: Il n'est pas nécessaire d'avoir une information courte alors que l'information longue ne fait que 6 pages.

Commentaire [CER2]: Ce terme peut être choquant: remplacer par….patients ayant souffert d'un cancer infantile. S'applique à toutes les sections où il y a lieu de le faire.

Commentaire [CER3]: Le contenu de ce chapitre n'est pas clair car il semble concerner à la fois les "outpatients" et les "inpatients", Suggestion: distinguer les deux groupes

Commentaire [CER4]: Pas clair : de quelle lettre s'agit-il et qui est le médecin personnel ?

Commentaire [CER5]: Si le questionnaire est complété dans le cadre de l'étude, veuillez indiquer le temps approximatif dont les participants auront besoin pour le compléter.

Commentaire [CER6]: De quel médecin s'agit-il?

décidez plus tard de vous retirer de l'étude, les données et le matériel en question seront détruits.	
**Vos obligations si vous participez à l'étude:**<br>Si vous décidez de participer à l'étude, vous devrez observer certaines règles pour votre propre sécurité. Si un échantillon d'urine vous est demandé lorsque vous êtes à la maison ou lors d'un séjour comprenant une nuit à l'hôpital, nous vous prions de collecter votre urine immédiatement à votre réveil. Au cas où vous êtes invité à fournir un échantillon d'urine durant une visite clinique, nous vous demandons de nous fournir un échantillon de votre urine au réveil, à la maison, et de l'apporter à l'hôpital.	5
**Ce qu'il adviendra de vos données:**<br>Nous respectons toutes les dispositions légales relatives à la protection des données. Nous utiliserons vos données uniquement dans le cadre de l'étude. Toutes les personnes impliquées sont soumises au secret professionnel.	5
**Ce que votre consentement signifie:**<br>Cette information courte est suivie d'informations complémentaires détaillées. Celles-ci font partie intégrante de la feuille d'information destinée aux participants à l'étude. En signant la déclaration de consentement, vous déclarez accepter l'intégralité du document.	7/8
**La personne à qui vous pouvez vous adresser:**<br>Vous pouvez poser toutes vos questions et demander toutes les précisions nécessaires à la personne suivante:<br>Nom: Dr. Med. Maja Beck Popovic<br>Fonction: Cheffe de projet<br>Adresse: Rue du Bugnon 46, 1011, Lausanne<br>numéro de téléphone: 021-314 35 90	5

1

Feuille d'information pour les participants

**Sommaire**		Page 3 sur 6
1	Sélection des personnes pouvant participer à l'étude	3
2	Objectifs de l'étude	3
3	Informations générales sur l'étude	3/4
4	Déroulement pour les participants	4
5	Droits des participants	4/5
6	Obligations des participants	5
7	Bénéfices pour les participants	5
8	Risques et contraintes pour les participants	5
9	Confidentialité des données et des échantillons	5/6
10	Exploitation ultérieure des données et des échantillons biologiques	6
11	Rémunération des participants	6
12	Financement de l'étude	6
13	Interlocuteur(s)	6

Titre de l'étude
SCCSS Etude pilote: collecte d'urine fraîche auprès de survivants de cancer infantile, afin d'évaluer l'impact de la consommation alimentaire

Cette étude est organisée par: Ligue Suisse contre le Cancer

Madame, Monsieur,

Je m'appelle Dr. Maja Beck Popovic et suis responsable de l'étude «Collecte d'urine fraîche auprès de survivants de cancer infantile»

1. Sélection des personnes pouvant participer à l'étude

La participation est ouverte à toutes les personnes qui ont souffert d'un cancer durant leur enfance et qui sont âgées de plus de 16 ans.
~~Elle est en revanche fermée aux personnes qui souffrent de maladies rénales terminales. Sont également exclues de l'étude les femmes enceintes.~~

Commentaire [CER7]: Supprimer car on ne proposera pas aux femmes enceintes et aux personnes qui souffrent de maladies rénales terminales de participer à cette étude

2. Objectifs de l'étude

Cette étude doit nous permettre d'établir des liens entre les résultats de vos analyses d'urine et le questionnaire auquel vous répondrez au sujet de votre consommation alimentaire.

Commentaire [CER8]: Expliquer brièvement comment sera établi le lien.

Commentaire [CER9]: Certains des "inpatients" n'ont-ils pas déjà répondu?

3. Informations générales sur l'étude

Vous avez récemment participé à une enquête menée dans le cadre de la Cohorte Suisse de Survivants de Cancer Infantile. Lorsque vous avez participé ~~part~~ à cette enquête, nous vous avons demandé quelles avaient été vos habitudes alimentaires durant le dernier mois. Aujourd'hui, nous

vous écrivons afin de vous demander de bien vouloir nous fournir un échantillon d'urine. Cet échantillon d'urine nous apportera des informations importantes relatives en lien avec votre consommation alimentaire évaluée par le questionnaire précédemment rempli. La collecte d'urine ne représente aucun danger et n'engendre aucune gêne. En effectuant une collecte d'urine, nous espérons en apprendre plus au sujet de la consommation alimentaire des survivants d'un cancer infantile. Ces informations seront utilisées dans la recherche sur les conséquences à long terme des traitements contre le cancer Nos recherches contribueront à réduire et, si possible, éviter ces conséquences à long terme du traitement.

Cette étude restreinte avec collecte d'échantillons menée par le CHUV, Lausanne, va donner un premier aperçu sur la question. Elle durera 12 mois et ~~nécessitera~~ concernera 200 participants. Les résultats serviront éventuellement à planifier une étude au niveau national.

Nous effectuons cette étude dans le respect des prescriptions de la législation suisse. Nous suivons en outre l'ensemble des directives reconnues au niveau international. La commission cantonale d'éthique compétente a contrôlé et autorisé l'étude. ~~Vous trouverez aussi un descriptif de l'étude sur le site Internet de l'Office fédéral de la santé publique: www.kofam.ch; le numéro de registre de l'étude est le: xxx~~

Commentaire [CER10]: Il n'y a pas d'enregistrement prévu pour les études ORH.

4. Déroulement pour les participants

~~Si vous êtes toujours suivi, vous serez recruté par la cheffe de projet, qui est également votre médecin traitant. Une lettre d'information vous demandant votre accord de participation vous parviendra ou vous sera remise personnellement.~~ Si vous êtes toujours en traitement au CHUV, l'étude vous sera proposée par votre médecin de l'étude qui vous remettra également le formulaire d'information. Après signature ~~d'une attestation de~~d'un consentement éclairé, un kit de collecte d'urine vous parviendra à votre domicile afin de récolter un échantillon de première urine matinale. Vous pourrez remettre cet échantillon directement à votre médecin traitant lors de votre visite clinique. Si vous devez séjourner à l'hôpital, le kit vous sera remis le jour précédant votre entrée, afin de collecter votre première urine matinale. Une fois collecté, votre échantillon d'urine sera remis aussi rapidement que possible au laboratoire du CHUV, pour analyse.

Si vous ne consultez plus votre médecin régulièrement, vous avez reçu par courrier ~~cette lettre~~ce formulaire d'information et une lettre de consentement sollicitant votre participation. Si vous acceptez de participer le kit de collecte d'urine sera expédié par le CHUV à votre adresse de domicile, afin de collecter un échantillon de première urine matinale. Une fois l'échantillon collecté, vous êtes prié de l'envoyer dans un délai de 24 heures aux laboratoires du CHUV, pour analyse.

Commentaire [CER11]: De quel médecin s'agit-il?

Si vous acceptez de participer, d~~D~~es informations concernant votre prise de médicaments et le type de traitement que vous suivez seront extraites de votre dossier médical. De plus, on vous demandera de transmettre, avec le kit de collecte d'urine, votre taille, votre poids, votre sexe (à des fins de vérification) si vous suivez un régime alimentaire personnalisé, vous avez des intolérances ou allergies alimentaires

5. Droits des participants

Vous devez prendre part à cette étude uniquement selon *votre* propre volonté. Personne n'est en droit de vous y pousser ou de vous influencer de quelque manière que ce soit. Si vous choisissez

de ne pas participer, votre traitement médical actuel se poursuivra exactement de la même manière. Vous n'aurez pas à justifier votre refus. Si vous choisissez de participer, vous pourrez à tout moment revenir sur cette décision. Là non plus, vous n'aurez pas à justifier votre retrait de l'étude.
Vous pouvez à tout moment poser toutes les questions nécessaires au sujet de l'étude. Veuillez-vous adresser pour ce faire à la personne indiquée à la fin de la présente feuille d'information.

6. Obligations des participants

Si vous décidez de participer à l'étude, vous serez tenu de suivre les instructions médicales de votre médecin-investigateur ~~et de vous conformer au plan de l'étude~~. Au cas où vous serez invité à fournir un échantillon d'urine lors de votre visite clinique, nous vous demanderons d'apporter votre échantillon de première urine matinale avec vous. Un kit de collecte d'urine vous sera envoyé à votre domicile. Nous vous demandons de collecter un échantillon directement à votre réveil, le jour de la visite clinique prévue. Si un échantillon d'urine est demandé durant un séjour comprenant une nuit à l'hôpital, nous vous demandons de collecter l'échantillon immédiatement au réveil. Dans les deux cas, vous pouvez transmettre votre échantillon d'urine à votre médecin traitant ou au personnel soignant. Si un échantillon de première urine au réveil vous est demandé lorsque vous êtes à votre domicile, il faut l'envoyer le jour même à l'hôpital. Nous vous fournirons tout le matériel d'envoi. Aucun frais d'envoi ne sera à votre charge. L'adresse de l'hôpital sera déjà mentionnée sur le colis prépayé.

Commentaire [CER12]: Confusion entre médecin traitant et médecin de l'étude

7. Bénéfices pour les participants

Si vous participez à l'étude, les résultats de l'étude pourraient se révéler utiles par la suite pour les personnes qui seront touchées par un cancer. Cette étude participera au développement de stratégies nutritionnelles et recommandations pour les survivants à un cancer infantile.

Commentaire [CER13]: Il doit d'abord être indiqué, qu'il n'y aura pas de bénéfices directs pour les participants.

8. Risques et contraintes pour les participants

Les participants n'encourent aucun risque dans le cadre de cette étude. La collecte d'urine est une méthode non-invasive.

9. Confidentialité des données et des échantillons

Commentaire [CER14]: Veuillez également spécifier le devenir des données collectées après la fin de l'étude

Nous serons amenés, pour les besoins de l'étude, à enregistrer vos données personnelles et médicales ainsi qu'un échantillon biologique. Toutefois, nous coderons ces données et échantillons. Le codage signifie que toutes les données permettant de vous identifier (p. ex. le nom, la date de naissance, etc.) sont remplacées par un code, de sorte que les personnes ne connaissant pas ce code ne peuvent pas lier ces données à votre personne. Au sein de l'Institut de Médecine Sociale et Préventive (ISPM) à Berne, les données et échantillons peuvent être consultées par les personnes autorisées et clairement désignées, y compris sous une forme non codée. Le code reste en permanence au sein de l'institution.

Commentaire [CER15]: Préciser qui codera les données, qui le conservera et qui y aura accès.

Commentaire [CER16]: Pourquoi à Berne?

Durant son déroulement, l'étude peut faire l'objet d'inspections. Celles-ci peuvent être effectuées par les autorités qui se sont chargées de son contrôle initial et l'ont autorisée, mais aussi être mandatées par l'organisme qui l'a initiée. Leur objectif est de s'assurer que les règles en vigueur sont bien respectées et que votre sécurité n'est pas menacée. Il se peut que le directeur de l'étude doive communiquer vos données personnelles et médicales pour les besoins de ces inspections.

Toutes les personnes impliquées dans l'étude de quelque manière que ce soit sont tenues à une confidentialité absolue. Nous ne ferons apparaître votre nom dans aucun rapport ou publication imprimé ou en ligne.

Il se peut que les données et échantillons liés à votre santé soient ultérieurement envoyés à des fins d'analyse dans le cadre de cette étude, à une autre biobanque située en Suisse. Celle-ci doit toutefois obéir aux mêmes normes et exigences que la biobanque de la présente étude.

> **Commentaire [CER17]:** Laquelle ? Il n'a pas été expliqué au participant qu'une partie de l'urine a été collectée pour la biobanque. Distinguer ce qui est collecté pour analyse dans le cadre de l'étude et pour la biobanque. Indiquer le but des deux biobanques

10. Exploitation ultérieure des données et des échantillons biologiques

Vous pouvez à tout moment vous retirer de l'étude si vous le souhaitez. Toutes les données vous concernant seront détruites, ainsi que le matériel biologique stocké.

> **Commentaire [CER18]:** En principe les données recueillies jusqu'au retrait de l'étude doivent être exploitées, notamment pour éviter des biais avant d'être anonymisées ou détruites si le participant en fait la demande.

11. Rémunération des participants

Il n'est prévu aucune rémunération pour la participation à cette étude.

12. Financement de l'étude

L'étude est majoritairement financée par Ligue Suisse contre le Cancer.

> **Commentaire [CER19]:** Et par qui d'autre?

13. Interlocuteur(s)

En cas de doute ou de question, vous pouvez vous adresser à l'un des interlocuteurs suivants :

Directeur de l'étude: Dr. Med. Maja Beck Popovic
Adresse: Rue du Bugnon 46, 1011 Lausanne
Numéro de téléphone: 021-314 35 90

Collaborateur: Angéline Chatelan
Adresse: Route de la Corniche 10, 1010 Lausanne
Numéro de téléphone: 021-314 82 38

Consentement éclairé écrit du participant pour la participation à une étude observationnelle

Déclaration de consentement écrite pour la participation à une étude

- Veuillez lire attentivement ce formulaire.
- N'hésitez pas à poser des questions lorsque vous ne comprenez pas quelque chose ou que vous souhaitez avoir des précisions.

**Numéro de l'étude**:	Référence numéro: xxx<br>Commission d'éthique de la recherché clinique vaudoise<br>Secrétariat central de la commission cantonale d'éthique de la recherche sur l'être humain<br>Avenue de Chailly 23, 1012 Lausanne
**Titre de l'étude:**	SCCSS Pilot study: urine spot collection among childhood cancer survivors
**Institution responsable (promoteur):**	Centre Hospitalier Universitaire Vaudois (CHUV), Service et laboratoire d'hématologie<br>BH 11 913, Rue du Bugnon 46, 1011 Lausanne<br><br>Ligue Suisse contre le Cancer/ Krebsliga Schweiz<br>Effingerstrasse 40, Postfach, 3001 Bern
**Lieu de réalisation de l'étude:**	Lausanne
**Directeur / directrice de l'étude sur le site:**	Dr. Med. Maja Beck Popovic
**Participant / participante:**<br>**Date de naissance:**	☐ femme ☐ homme

- Je déclare avoir été informé(e), par le médecin/responsable de l'étude soussigné, oralement et par écrit, des objectifs et du déroulement de l'étude concernant une collecte d'urine fraîche, ~~ainsi que des effets présumés~~, des avantages, des inconvénients possibles et des risques éventuels.
- J'ai reçu des réponses satisfaisantes aux questions que j'ai posées en relation avec ma participation à l'étude. Je conserve la feuille d'information datée du 14 décembre 2015 ~~/ dans sa version 1 (deux parties)~~ et reçois une copie de ma déclaration de consentement écrite. J'accepte le contenu de la feuille d'information qui m'a été remise sur l'étude précitée.
- Je prends part à cette étude de façon volontaire. Je peux, à tout moment et sans avoir à me justifier, révoquer mon consentement à participer à l'étude, sans que cela n'ait de répercussion défavorable sur la suite de ma prise en charge médicale.
- J'ai eu suffisamment de temps pour prendre ma décision.
- Je sais que mes données personnelles et échantillons biologiques peuvent être transmis à des fins de recherche dans le cadre de cette étude uniquement sous une forme codée. J'accepte que les spécialistes compétents du mandataire de l'étude, ~~des autorités et de la commission d'éthique compétente~~ pour cette étude puissent consulter mes données brutes afin de

Commentaire [CER20]: A compléter

Commentaire [CER21]: Une information orale est-elle prévue pour les outpatients ?

Commentaire [CER22]: Pas pertinent car il ne s'agit pas d'une étude thérapeutique

Commentaire [CER23]: Y a-t-il des avantages, inconvenients et risques? Si oui, en faire mention dans le formulaire d'information. Si non, supprimer.

Commentaire [CER24]: A qui? Préciser s'il s'agit d'une biobanque

procéder à des contrôles, à condition toutefois que la confidentialité de ces données soit strictement assurée.

- J'accepte que mes données et échantillons biologiques soient réutilisés dans le cadre d'autres études: oui ☐ non ☐
- Je désire que mon médecin traitant soit informé des résultats de cette étude oui ☐ non ☐
- Je suis conscient(e) que les obligations mentionnées dans la feuille d'information destinée aux participants doivent être respectées pendant la durée de l'étude.

Commentaire [CER25]: Préciser….. études dans le domaine de la nutrition et le cancer qui seront préalablement soumis à la Commission d'éthique pour approbation

Lieu, date	Signature du participant / de la participante

Attestation du médecin-investigateur: Par la présente, j'atteste avoir expliqué au participant / à la participante la nature, l'importance et la portée de l'étude. Je déclare satisfaire à toutes les obligations en relation avec cette étude conformément au droit en vigueur. Si je devais prendre connaissance, à quelque moment que ce soit durant la réalisation de l'étude, d'éléments susceptibles d'influer sur le consentement du participant / de la participante à prendre part à l'étude, je m'engage en l'en informer immédiatement.

Commentaire [CER26]: Comment va-t-on obtenir le consentement des « outpatients » ?

Lieu, date	Signature du médecin-investigateur: Dr. Med. Maja Beck Popovic
